# Easy regulation of metabolic flux in *Escherichia coli* using an endogenous type I-E CRISPR-Cas system

**DOI:** 10.1186/s12934-016-0594-4

**Published:** 2016-11-15

**Authors:** Yizhao Chang, Tianyuan Su, Qingsheng Qi, Quanfeng Liang

**Affiliations:** State Key Laboratory of Microbial Technology, Jinan, 250100 China

**Keywords:** Type I-E CRISPR-Cas system, TCA cycle, *Escherichia coli*, Poly-3-hydroxbutyrate

## Abstract

**Background:**

Clustered regularly interspaced short palindromic repeats interference (CRISPRi) is a recently developed powerful tool for gene regulation. In *Escherichia coli*, the type I CRISPR system expressed endogenously shall be easy for internal regulation without causing metabolic burden in compared with the widely used type II system, which expressed dCas9 as an additional plasmid.

**Results:**

By knocking out *cas*3 and activating the expression of CRISPR-associated complex for antiviral defense (Cascade), we constructed a native CRISPRi system in *E. coli*. Downregulation of the target gene from 6 to 82% was demonstrated using green fluorescent protein. Regulation of the citrate synthase gene (*gltA*) in the TCA cycle affected host metabolism. The effect of metabolic flux regulation was demonstrated by the poly-3-hydroxbutyrate (PHB) accumulation in vivo.

**Conclusion:**

By regulating native *gltA* in *E. coli* using an engineered endogenous type I-E CRISPR system, we redirected metabolic flux from the central metabolic pathway to the PHB synthesis pathway. This study demonstrated that the endogenous type I-E CRISPR-Cas system is an easy and effective method for regulating internal metabolic pathways, which is useful for product synthesis.

**Electronic supplementary material:**

The online version of this article (doi:10.1186/s12934-016-0594-4) contains supplementary material, which is available to authorized users.

## Background

The ability to precisely manipulate expression level of the desired genes by repression or activation is important for understanding the complex functions of a gene network. RNA interference (RNAi) and engineered DNA-binding proteins are powerful technologies for gene regulation [1–3]. RNAi can be employed to knock down the expression of targeted genes. However, RNAi is limited to particular organisms that have the proper host machinery and can sometimes exhibit significant off-target effects and toxicity [3]. In addition, custom DNA-binding proteins, such as transcription-activator-like effector (TALE) proteins or zinc finger, remain somewhat difficult and expensive to design, develop, and empirically test in the cellular context [1, 2].

The CRISPR-Cas system is an antivirus mechanism among Archaea and Bacteria [4–6]. Since the verification of its function in cutting DNA and first use in gene editing [7, 8], this system has been widely used in various areas of research [9–13]. CRISPRi is one of these utilities. By mutating the DNase domain of Cas9 (class 2 type-II) or removing Cas3 (class 1 type-I) manually, the CRISPR system is inactivated for its DNA-cutting function, with DNA-binding function maintained [8, 11, 14]. This enables the system to bind to DNA without further cutting it and thus impedes transcription, which facilitates the ability to regulate gene expression and is known as CRISPRi [11, 15]. Because of the advantages of CRISPRi-a system requiring only Cas proteins and a single guide RNA (sgRNA) or CRISPR RNA (crRNA), with abundant targeting sites on the genome, being easy for targeting multi-genes with relatively low off-target potential and showing reversible regulation effects [9, 11, 13, 16], it has been used in a variety of species.

**Fig. 1:**
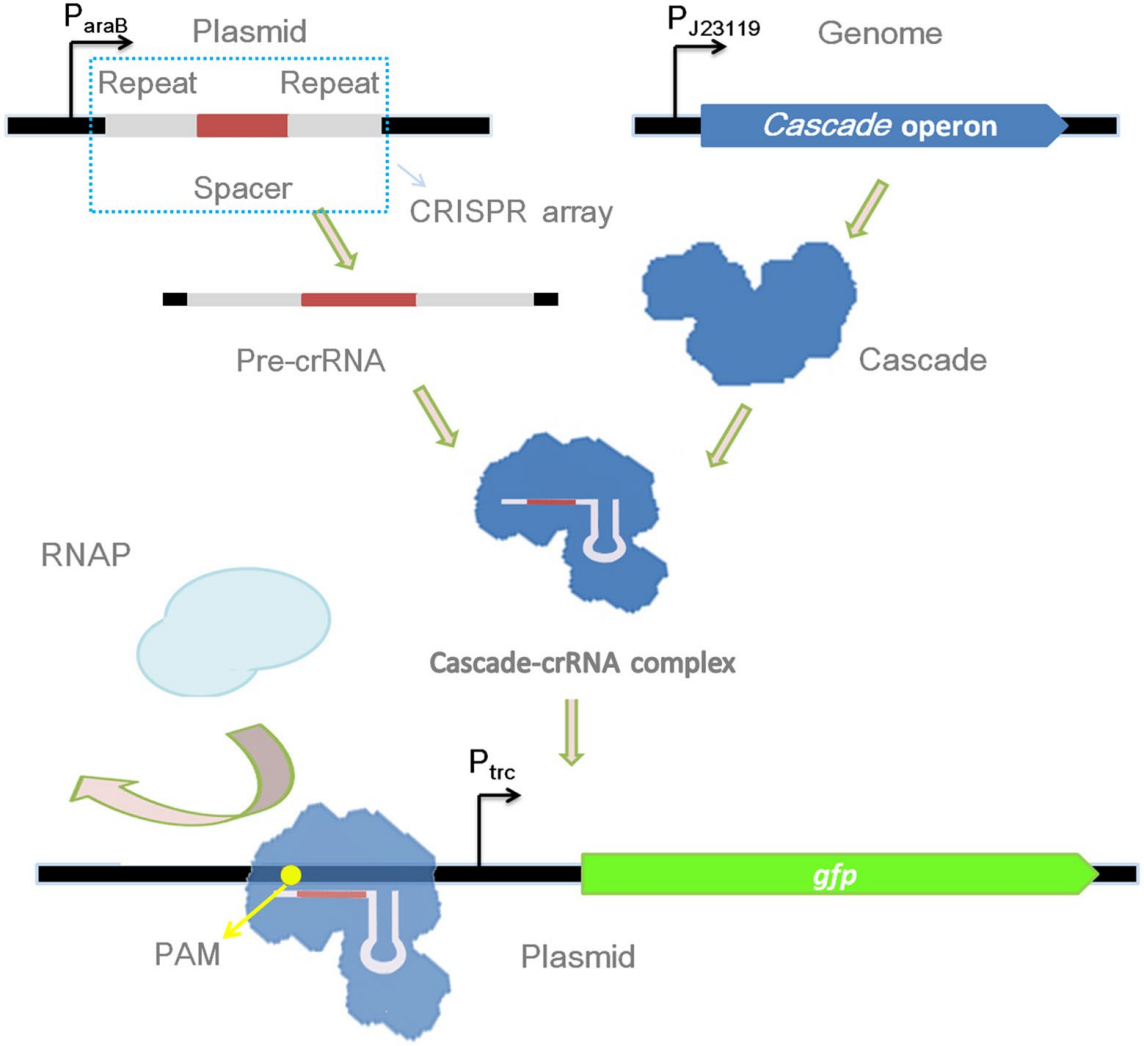
CRISPRi process used in this study. The crRNA was expressed on a plasmid and Cascade was activated for expression with the constitutive promoter J23119. Cascade mediates the maturation of crRNA and forms complex with the crRNA. The complex then binds to a target site to disturb transcription. RNAP indicates RNA polymerase, PAM indicates protospacer adjacent motif

Currently, class 2 type-II CRISPRi is the most widely used CRISPR system. Apart from sgRNA, this system need additionally express dCas9 (4 Kb), which requires the expression of a second vector in *Escherichia coli* [12]. Recently, two studies reported that endogenous class 1 type I-E CRISPR system could be repurposed for gene regulation [16, 17]. Typically, in class 1 type I, the Cascade mediates the maturation of crRNA and forms complex with it, which then binds to the target site on DNA and recruits Cas3 to degrade the target DNA [7, 18–21]. By knocking out the *cas*3 gene in the genome, the Cascade-crRNA complex retains the ability to bind to DNA [16, 17], functioning as a transcription regulation factor (Fig. 1). This system only requires the engineering of the strain and the expression of the CRISPR array for gene regulation purpose, and thus can be easily used for internal regulation without causing a metabolic burden. However, the endogenous type I-E CRISPR-Cas system has not been employed for biotechnological applications in *E. coli*.

Phosphoenolpyruvate, pyruvate, and acetyl-CoA are precursors for both the tricarboxylic acid cycle (TCA) and most synthetic pathways. Therefore, the production of these chemicals competes with the TCA cycle. To improve biochemical production from a desired synthetic pathway, genes encoding the enzymes for competing pathways are often knocked out [22]. However, the deletion of genes associated with the TCA cycle has negative effects on cell growth and final cell density, and these genes are rarely employed as the deletion candidate target to increase the titer and yield of a target compound [23].

Here, after engineering the endogenous CRISPR system, the metabolic effect of *gltA* regulation was evaluated. PHB production in *E. coli* was chosen as a model to demonstrate the redirection of metabolic flux. Our results showed that the endogenous type I-E CRISPR-Cas system is an easy and effective method that can be used to regulate metabolic pathways.

## Results

### Construction and characterization of an endogenous CRISPRi system in *E. coli*

To construct the *E. coli* endogenous CRISPRi for gene regulation, we first disabled its DNA degradation function and maintained its DNA binding function by substituting *cas*3 and the promoter of the Cascade operon with the constitutive promoter J23119 in *E. coli* TOP10 through homologous recombination (Additional file 1: Figure S1). The resulting strain TOP10Δ*cas*3 was verified by PCR and agarose gel electrophoresis. Plasmid pcrRNA.*Bbs*I was constructed and transformed into the strain to facilitate crRNA expression (Additional file 1: Figure S2).

**Fig. 2:**
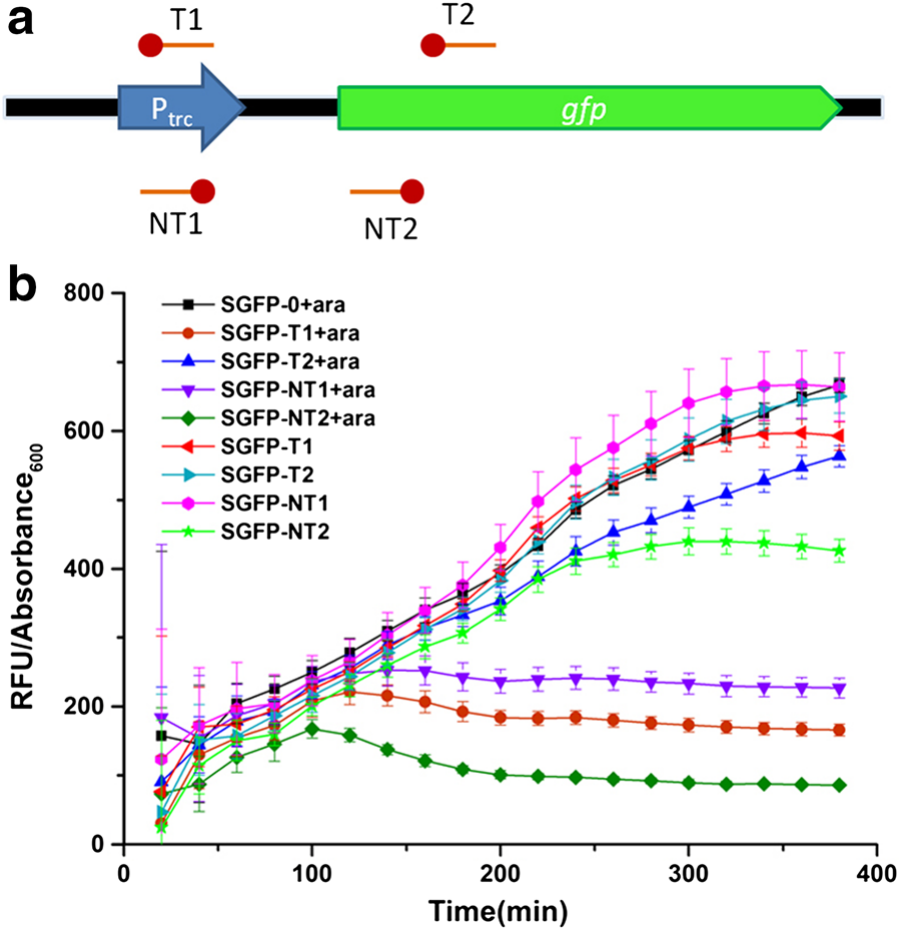
Verification of endogenous CRISPRi using GFP. **a** Spacers designed to target *gfp* on the plasmid. The *red circles* indicate the PAM sequence; *red lines* indicate the spacer. **b** Endogenous CRISPRi repressed *gfp* expression. The strains were cultured in a 96-well plate in 200 μL LB medium, with or without 0.2% l-arabinose. The *error bars* indicates the standard deviations of eight biological replicates. The 0 indicates the control and T1, T2, NT1, and NT2 indicate the corresponding spacer names

Next, to verify the function of the system, a plasmid expressing GFP (PLYK) was co-transformed with crRNA expression vectors PGFP-Y into *E. coli* TOP10Δ*cas*3 to construct series strains SGFP-Y (Y indicates 0, T1, T2, NT1, and NT2, which are spacer names; 0 indicates control, targeting no sites). Spacers were designed using a self-designed program to avoid potential off-target effects (Fig. 2a; Additional file 1: Figure S3). As demonstrated in other studies [16, 17], targeting different sites of the gene can lead to different regulation effects. We observed a wide range of fluorescent repression (from 6 to 82%) among the spacers (Fig. 2b). In the presence of l-arabinose, the spacers (T1, NT1) targeting the promoter region of both strands showed strong repression effects, while T2 (targeting template strand) and NT2 (targeting non-template strand) showed the lowest (6%) and highest (82%) repression levels, respectively.

### Optimizing expression of crRNAs

**Fig. 3:**
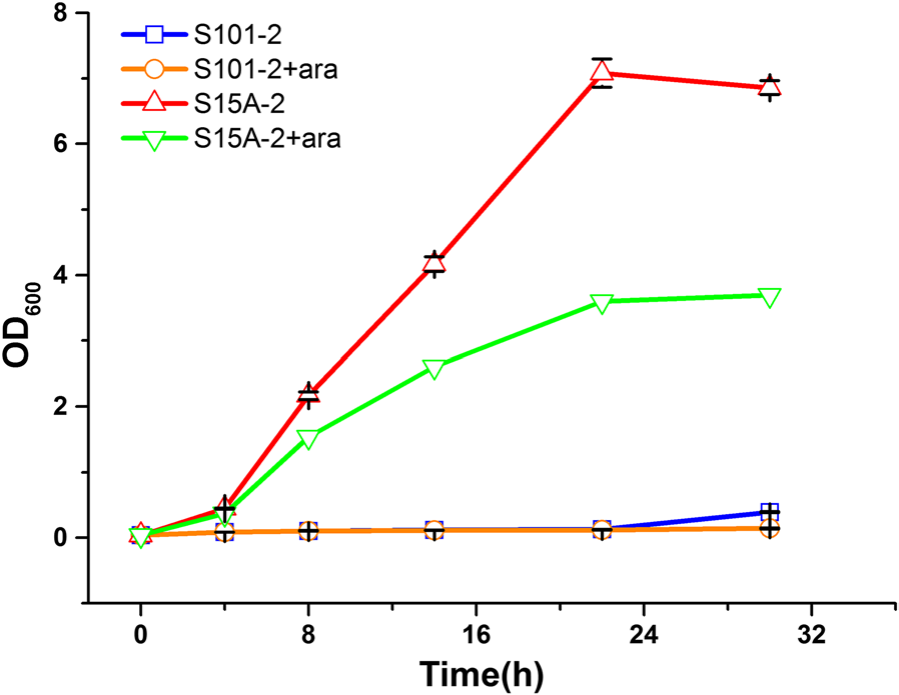
Comparison of the regulation effects between low-copy and medium-copy plasmids. Strains were cultured in 50 mL M9 medium containing 1% (v/v) glycerol and 0.2% (g/v) l-arabinose added at 0 h. The *error bars* indicate the standard deviations of three biological replicates

**Fig. 4:**
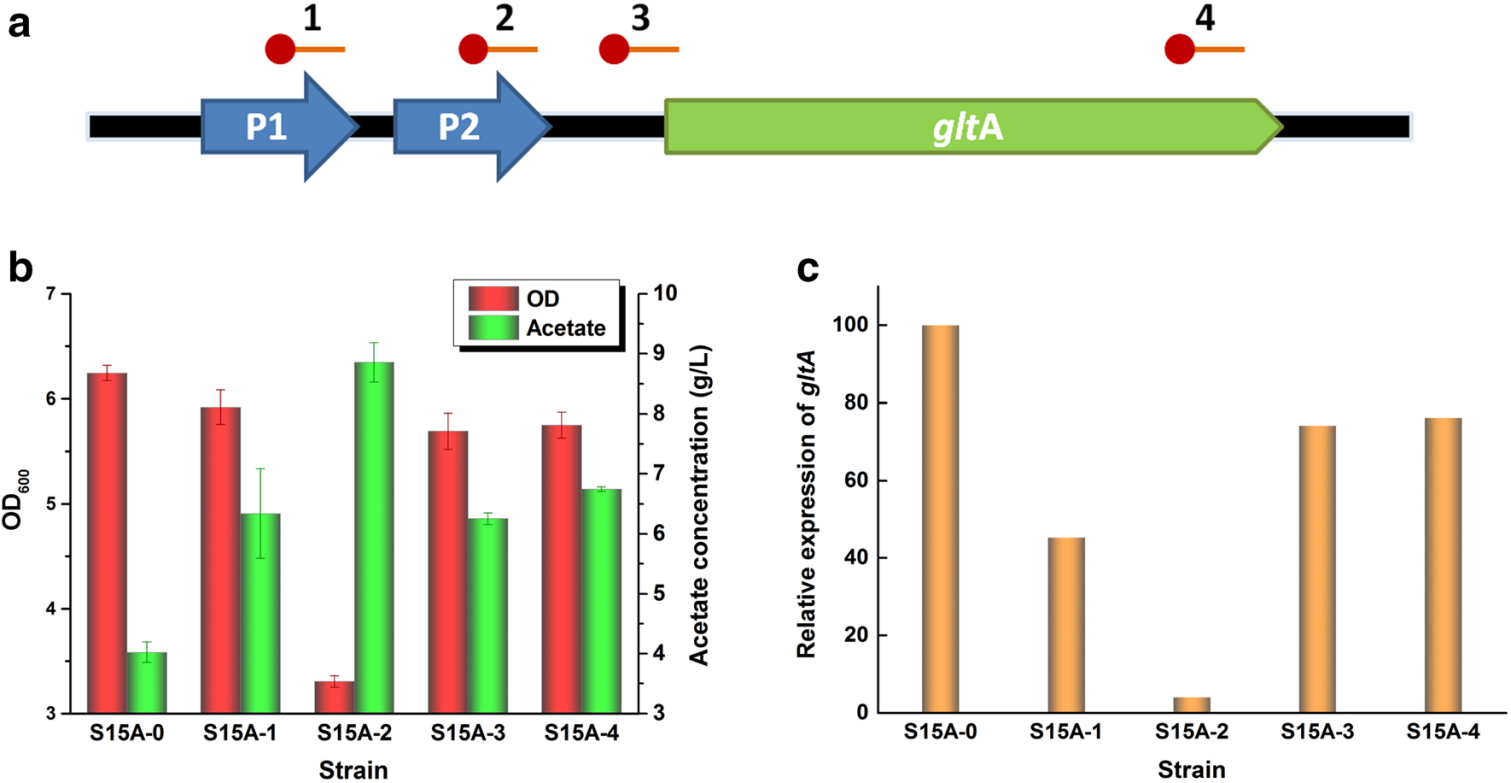
Targeting at different sites on *gltA* using endogenous CRISPRi. **a** Spacers targeting *gltA* on the genome. The *red circles* indicate the PAM sequence and *red lines* indicate the spacer. P1 and P2 indicate the two promoters for native *gltA*. **b** Cell growth and acetate accumulation by S15A-N. **c** Transcription variances among different strains with *gltA* targeted at different sites. The expression of S15A-0 was set to 100, while expression of other strains was calculated relative to this value. Strains were cultured in 50 mL M9 medium containing 1% (v/v) glycerol and 0.2% (g/v) l-arabinose added at 0 h. The *error bars* indicate the standard deviations of three biological replicates

The TCA cycle is one of the most important processes in central metabolism. It begins with the formation of citric acid from acetyl-coA and oxaloacetate, which is catalyzed by citrate synthase [24]. This process is irreversible and is the rate-limiting step in the TCA cycle. The regulation of *gltA*, which encodes citrate synthase, will affect the TCA cycle, and thus regulate the metabolic flux of central metabolism. To optimize the expression of crRNAs in *E. coli*, we first constructed a low-copy plasmid (Paracr101) and medium-copy plasmid (Paracr15A) to express crRNAs with spacers targeting endogenous *gltA* in TOP10Δ*cas*3, while a high copy plasmid was used for product synthesis. A spacer targeting the latter one of the two promoters of *gltA* was used to compare the two plasmids [25], which was designed to have tight repression effects. The strains containing the medium-copy plasmid (S15A-2) or low copy plasmid (S101-2) showed little growth variance when cultured in LB medium (Additional file 1: Figure S4). Next, both strains were cultured in 50 mL M9 medium. As shown in Fig. 3, the two strains showed significant variance in growth. For S15A-2, decreased growth was observed when l-arabinose was provided. For S101-2, growth was poor with or without l-arabinose. This indicated that the expression of crRNAs could not be harnessed by adding inducer using a low copy number plasmid when the strain was cultured in M9 medium. Therefore, the medium copy plasmid for crRNA expression was used in subsequent analyses. The spacers used were designed by the self-designed program as described above to avoid potential off-target effects (Fig. 4a).

### Down-regulation of *gltA* at different levels using endogenous CRISPRi

To investigate the regulation effects of targeting *gltA* at different sites, we constructed a series of strains S15A-N (N indicates 0, 1, 2, 3, 4) containing spacers of the corresponding number for fermentation (Fig. 4a). As shown in Fig. 4b, all strains showed repressed growth compared to the control, with strain S15A-2 showing strongest repression. For the accumulation of acetate, all strains produced more acetate than the control, with S15A-2 producing the highest concentration of 8.85 g/L. When both promoters of *gltA* were targeted, targeting the latter strongly repressed the growth of the strains and increased the accumulation of acetate. The transcription of *gltA* among the strains was also analyzed by qRT-PCR. Method of relative quantification with standard curve was used (Additional file 1: Figure S5). The expression of *gltA* was down-regulated from 1.5- to 25-fold (Fig. 4c), with S15A-2 showing the strongest repression, which was in accordance with the growth results described above. As S15A-3 and S15A-4 showed little difference in growth, acetate accumulation and *glt*A transcription, we chose the spacer4 to construct strains in following studies.

Furthermore, we investigated the regulation effects of targeting *gltA* at different induction times by adding l-arabinose at 0, 12 and 24 h to strains S15A-X (X indicates 0, 1, 2, 4). As shown in Table 1, all strains grew better when l-arabinose was added at 12 or 24 h compared to addition at 0 h. Glycerol consumption was correlated with growth. All strains showed growth repression when l-arabinose was added at 0 h, including the control (Additional file 1: Figure S6). This may be because l-arabinose is toxic to these strains [26].

**Table 1:** Cell growth, glycerol consumption and acetate accumulation at different induction times

Content	Induction time (h)	Strain^a^			
		S15A-0	S15A-1	S15A-2	S15A-4
OD_600_^b^	0	5.93 ± 0.68	6.45 ± 0.48	3.45 ± 0.04	5.22 ± 0.44
	12	7.02 ± 0.15	6.63 ± 0.35	6.43 ± 0.10	6.53 ± 0.22
	24	6.61 ± 0.13	6.82 ± 0.18	6.23 ± 0.12	6.94 ± 0.33
Glycerol^b^ (g/L)	0	12.89 ± 0.00	12.89 ± 0.00	9.78 ± 0.68	12.52 ± 0.46
	12	12.62 ± 0.31	12.26 ± 0.90	12.22 ± 0.12	12.60 ± 0.41
	24	12.89 ± 0.00	12.89 ± 0.00	12.89 ± 0.00	12.89 ± 0.00
Acetate^b^ (g/L)	0	2.65 ± 0.55	2.84 ± 0.22	3.81 ± 0.68	3.48 ± 0.52
	12	3.28 ± 0.35	3.95 ± 0.50	3.36 ± 0.20	4.09 ± 0.21
	24	3.40 ± 0.18	3.66 ± 0.22	3.22 ± 0.64	3.34 ± 0.42

^a^Strains were cultured in 50 mL M9 medium containing 1% (v/v) glycerol and 0.2% (g/v) l-arabinose added 0 or 24 h

^b^Data are shown as the average values and standard deviations of two biological replicates, samples were taken at 42 h

### Demonstrating the redirection of metabolic flux using PHB accumulation

**Fig. 5:**
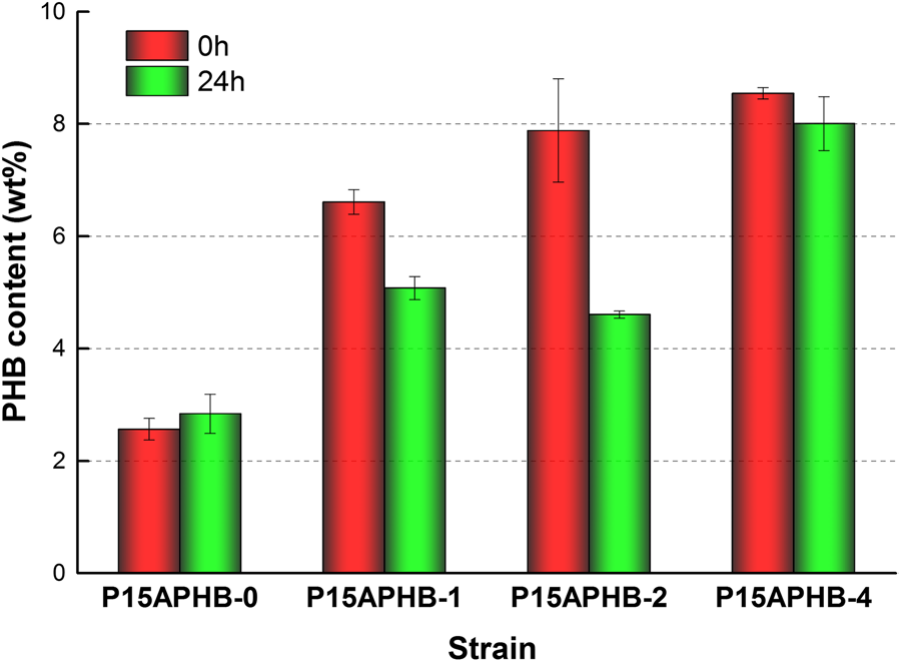
Production of PHB with *gltA* regulated using endogenous CRISPRi. Strains were cultured in 50 mL M9 medium containing 3% (v/v) glycerol. The inducer l-arabinose was added at 0 or 24 h at a concentration of 0.2% (g/v). The *error bars* indicate the standard deviations of three independent measurements

As proof of concept for the practical application of our regulation system, we introduced the PHB synthetic pathway to construct strain S15APHB-X. As shown in Additional file 1: Figure S7A, introduction of the PHB pathway eliminated the repression effect on growth, acetate accumulation among strains, which agrees with our previous results ([27], Additional file 1: Figure S7). Among the strains induced at 0 h, S15APHB-4 consumed the largest amount of glycerol and showed the highest PHB production (19.6 g/L and 8.5%, respectively), in which the PHB content was 3.4-fold higher than in the control (Fig. 5; Additional file 1: Figure S7B). When strains were induced at different time, all strains except for the control showed decreased PHB accumulation when induced at 24 h compared to at 0 h. (Fig. 5).

## Discussion

CRISPRi is a recently developed tool that can be used for transcription regulation [11]. Compared to the type II system, which was discovered in bacteria, type I is widely present in Bacteria and Achaea [28–30]. Numerous type I systems can be activated by deleting *cas*3, while others such as type I-A and type I-D can be used when Cas3 is mutated as dCas9 [16]. Using these systems, only crRNAs must be additionally expressed, which can be easy for regulation. Besides, most type I systems can recognize more PAM types than type II, which could enlarge the available targeting sites for regulation. Then the Cascade-bound R-loop is more stable than that of Cas9 [31, 32], enabling greater control over regulation. Additionally, dCas9 does not function properly in some Achaea, and thus an endogenous system is required for regulation purposes [33].

By using the engineered endogenous type I-E CRISPR system in *E. coli*, the effects in regulating GFP ranged from 6 to 82%. Spacers targeting the promoter region of both strands repressed GFP expression tightly, while targeting the non-promoter region on the non-template strand generally shows more repression effect than targeting the other strand [11, 16, 17, 33]. Interestingly, while transcription occurred on the template strand, targeting the non-template strand should cause a stronger repression effect. This might be related to the structural characteristics of RNA polymerase-DNA complex and Cascade-crRNA-DNA complex. The two promoters of native *gltA* were targeted using spacer1 and spacer2, respectively. However, only targeting the latter promoter had an significant repression effect on cell growth and transcription of *gltA*. This may be because when the former promoter was targeted, the latter could still function and thus was not tightly repressed.

The TCA is one of the most important processes in central metabolism. By regulating the expression of *gltA* using endogenous CRISPRi, the metabolism was redirected to PHB production. Before the introduction of PHB synthesis pathway, the repression level of *gltA* is nearly liner related to the growth and acetate accumulation of the strains, except for S15A-1. Recently, Soma et al. described a metabolic toggle switch with quorum sensing system as a sensor that can control metabolic flux from the TCA cycle towards the isopropanol synthetic pathway in appropriate time. The effects of switching *gltA* OFF on cell growth and acetate production were investigated [34, 35]. The inhibited levels of the *gltA* OFF strains growth decreased with increasing induction time, which was in accordance with our results of downregulating *gltA* by endogenous CRISPR-Cas system. However, after the introduction of PHB synthesis pathway, the variances on growth, acetate accumulation of strains containing different targeting sites were eliminated. The isopropanol production titer of the resulting strains was threefold higher than in the control strain [34, 35]. Our engineered *E. coli* produced three- to fourfold more PHB than the control strain.

Another common concern regarding the use of the CRISPR-Cas system is its off-target effects. A simple algorithm was developed to avoid potential off-target effects. In the regulation of GFP, spacer T1 was excluded by the program for 19 of its contiguous nucleotides and protospacer adjacent motif (PAM) are homologous to the genome (Additional file 1: Figure S8). To demonstrate the regulation effect of the promoter region, which no spacer was designed by the program, we designed spacer T1 manually and found that it off-targeted a pseudo gene with no known function in the genome. This fact proved that the GFP regulating result of this spacer was most likely caused by the regulation effect on the target site, but not a mixed result of targeting two sites on the genome, which proved that the result for spacer T1 could be used. In contrast to the well-studied type II system, for which numerous tools are available to predict off-target effects [36–40], few applicable tools exist for other types [41]. Then to decrease off-target phenomenon, the seed region of the spacer (with PAM) should be unique in the genome, which may be of vital importance for the base-pairing of sgRNA or crRNA with DNA [11, 20, 32, 40–45], or potential off-targeting sites of the spacer with no known functions should be confirmed.

## Conclusions

In this study, we developed an endogenous type I-E CRIPSRi system in *E. coli* by knocking out *cas*3. Using this simple regulation strategy, we redirected metabolic flux by downregulating *gltA* in TCA. Redirection of the metabolic flux was demonstrated using PHB accumulation, which increased by 3.4-fold compared to the control. This study demonstrated that the endogenous type I-E CRISPRi is easy and an effective method for regulating metabolic pathways.

## Methods

### Strain and plasmid construction

All strains and plasmids used in this study are shown in Additional file 1: Tables S1, S2. To reconstruct the endogenous type I-E CRISPR-Cas system, *E. coli* Top10Δ*cas*3 was generated through recombination by knocking out *cas*3 and substituting the native promoter of the Cascade operon with J23119 [16].

To express the crRNAs, plasmid pcrRNA.ind were digested with *Kpn*I and *Xho*I. The fragment was then ligated with annealed oligos *Bbs*I-f and *Bbs*I-r to form the plasmid pcrRNA.*Bbs*I.

To construct plasmids Paracr15A and Paracr101 expressing the crRNA, fragments of p15A-ori with spectinomycin (spc^R^) resistance (amplified from pLYK with cr15A-f and cr15A-r), pSC101-ori with spc^R^ resistance (amplified from PHBS01, cr101-f, cr101-r) were ligated to the backbone with *araC* CRISPR array (amplified from pcrRNA. *Bbs*I using 15Acr-f, 15Acr-r, and 101cr-f, 101cr-r, respectively), through Gibson-assembly.

To generate plasmids with the spacer targeting specific sites (PGFP-Y, Paracr15A-N, Paracr101-2), 1 μL pcrRNA.*Bbs*I and 1 μL annealed spacer pairs were added to a 30 μL mixture of 0.5 μL T4 DNA ligase, 3 μL T4 DNA ligase buffer (10×), 0.5 μL T4 PNK, 1 μL *Bbs*I, 0.2 μL bovine serum albumin, and 22.8 μL ddH_2_O. The PCR conditions were as follows: 25 °C for 10 min and 37 °C for 10 min for 15 cycles, 50 °C for 30 min, 80 °C for 30 min, and holding at 4 °C. The mixtures were then digested with 0.5 μL *Bbs*I and 0.5 μL plasmid safe ATP-dependent DNase at 37 °C for 30 min.

### Growth conditions

For strain and plasmid construction, strains were cultured in Luria–Bertani (LB) medium. For fermentation, strains were cultured in 50 mL M9 medium containing 2 g/L Amicase (Sigma, St. Louis, MO, USA), 0.2 g/L l-arabinose, and 1% (v/v) glycerol; 3% glycerol was used for PHB production. To maintain the plasmids, final concentrations of 100 μg/mL ampicillin, 50 μg/mL spectinomycin, and 25 μg/mL chloromycetin were added to the corresponding cultures.

### Spacer design

All protospacers used in this study are listed in the Additional file 1: Table S3. Spacers were selected by a self-designed algorithm considering GC content, poly-T structure, and seed (7–12 nucleotides in addition to PAM) together with PAM [17, 40] not homologous to other parts of the genome. The PAMs of AGG, ATG, and AAG, which had been proved to work were used.

### Fluorescence detection

Strains were pre-cultured in 5 mL LB medium in tubes overnight at 37 °C with shaking at 250 rpm. Next, 4 μL of the culture was added to 200 μL LB medium in a 96-well plate containing l-arabinose and the appropriate antibiotics. The plate was cultured and absorbance was measured using a microplate reader (Synergy HT, BioTek, Winooski, VT, USA) at 37 °C at a medium shaking speed to detect the fluorescence and cell density.

### qRT-PCR analysis

Fragments of *gltA* and 16S rRNA amplified from TOP10, together with fragments containing Amp^R^ and the pBR322 origin of replication were ligated through Gibson-assembly to construct plasmids PGLTA and P16S, respectively.

Total mRNA was extracted using an RNAprep Culture Cell/Bacterial Kit (Tiangen, Beijing, China). Next, 2 μL total mRNA was used for reverse transcription (cDNA synthesis) using the Primer Script RT reagent Kit with gRNA Eraser (TaKaRa, Shiga, Japan), and random primers were used according to the manufacturer instructions. Processes involving RNA and cDNA were conducted on ice except for reaction. The extracted RNA and cDNA were stored at −80 °C for no more than 2 weeks after density measurement.

Plasmid PGLTA and P16S were serially diluted to 10^2^, 10^4^, 10^6^, 10^7^, and 10^8^-fold (concentrations of 10^−2^, 10^−4^, 10^−6^, 10^−7^, 10^−8^, respectively) to construct the standard curves for the target (*gltA*) and internal control (16S). SYBR Premix Ex Taq™II (TaKaRa) was used for qPCR. The 25-μL reaction mixture contained 12.5 μL enzyme mix, 10 μL H_2_O, 0.5 μL ROX II, 0.5 μL forward primer (10 mM), 0.5 μL reverse primer (10 mM), and 1 μL sample cDNA or standard plasmid DNA. Primers RT-16S-for, RT-16S-rev, RT-GLTA-for, and RT-GLTA-rev were used to quantify *gltA* and 16S in the samples and standard, respectively. The primers were designed using Primer6 and the specificities were verified by additional qPCR. Three parallel reactions were conducted for each sample or standard. The reaction program was conducted using Quant Studio 3 (Thermo Fisher Scientific, Waltham, MA, USA) following the SYBR Premix Ex Taq™ II instructions. The specificity of qPCR was verified by melt-curve analysis of the amplified sequence. The results were analyzed using QuantStudio™ Design and Analysis software 1.3.1 automatically, and the exported results were analyzed using OriginPro 9.0 (Originlab, Northampton, MA, USA) [46, 47].

### Analysis of substrates and products

Biomass was measured as the optical density value at 600 nm using a spectrophotometer (Shimazu, Japan). To analyze acetate and glycerol, 1 mL of the culture was centrifuged at 12,000 rpm for 2 min; the supernatant was then filtrated through a 0.22-μm syringe filter and quantitatively examined by using high-performance liquid chromatography (HPLC) (Shimadzu, Japan) equipped with a refractive index detector (RID-10A) (Shimadzu, Japan) and an Aminex HPX-87H ion exclusion column (Bio-Rad, USA). A 5 mM H_2_SO_4_ solution was used as a mobile phase at a flow rate of 0.6 mL/min to the column at 65 °C. Standards were prepared for acetate and glycerol and calibration curves were created. The detection sensitivity was 0.1 μg compounds per HPLC assay (10 μL). The detection limit for the extracellular metabolites and carbon sources was 10 mg/L [48]. PHB was quantitatively analyzed using gas chromatography. Briefly, liquid cultures were centrifuged at 10,000*g* for 10 min, and then the cells were washed twice in saline and lyophilized overnight. About 15 mg lyophilized cell mass was mixed with 1 mL chloroform and 1 mL methanol containing 15% (v/v) sulfuric acid. The methanolysis was performed at 100 °C for 1 h in an oil bath. Then 1 mL water was added to the mixture and mixed thoroughly for 20 s. After phase separation, the heavier chloroform phase was transferred to another new vial for GC analysis. The PHB content was defined as the percentage ratio of the PHB concentration to biomass [27, 49].

### Genes and plasmids sequence

Genes and genome sequences were downloaded from NCBI [50].

## Additional file

**Additional file 1.** All strain’s name, plasmids, primers used in the study as well as figures mentioned in the main text are available in this file.

## Abbreviations

CRISPR: clustered regularly interspaced short palindromic repeat
CRISPRi: CRISPR interference
Cascade: CRISPR-associated complex for antiviral defense
*E. coli*: *Escherichia coli*
PHB: poly-3-hydroxbutyrate
TCA cycle: tricarboxylic acid cycle
RNAi: RNA interference
TALE: transcription-activator-like effector
sgRNA: single guide RNA
crRNA: CRISPR RNA
GFP: green fluorescent protein
qRT-PCR: quantitative reverse transcription polymerase chain reaction
PAM: protospacer adjacent motif
